# A Cohort of Sociodemographic and Health-Related Risk Factors for All-Cause Mortality in Middle-Aged and Older Adults in China

**DOI:** 10.3390/healthcare13172104

**Published:** 2025-08-24

**Authors:** Wenhu Xu, Hang Zhu, Yutian Chen, Qianyi Zhang, Zhinan Liu, Gong Chen

**Affiliations:** Institute of Population Research, Peking University, Beijing 100091, China; 2401111475@stu.pku.edu.cn (W.X.); chenyutian@stu.pku.edu.cn (Y.C.); zhangqianyi0124@163.com (Q.Z.); 2401213152@stu.pku.edu.cn (Z.L.)

**Keywords:** physical activity, mortality, aging, functional status, cohort study, Chinese older adults

## Abstract

**Background:** Physical inactivity is a major contributor to increased mortality among aging populations, especially in middle-aged and older adults. **Methods:** Data were derived from the China Health and Retirement Longitudinal Study (CHARLS, 2011–2020). Participants self-reported their physical activity frequency, categorized as low (≤1 day/week), medium (2–4 days/week), or high (≥5 days/week). All-cause mortality was tracked through verified records. Cox proportional hazards models were used to estimate hazard ratios (HRs), with adjustments for demographics, lifestyle factors, and baseline health conditions. **Results:** A total of 2092 participants (mean age = 63.7 ± 10.4 years) were included in the final analytic sample. Higher physical activity frequency was significantly associated with lower mortality in unadjusted models. Participants engaging in activity ≥5 days/week had a 67% reduced mortality risk compared to the low-frequency group (HR = 0.33, *p* < 0.001). However, after adjusting for health-related covariates, the protective effect was attenuated and no longer statistically significant. In the fully adjusted model, advanced age, current smoking, and ADL limitations emerged as the strongest independent risk factors for mortality, while being married and residing in a rural area were significantly protective effects. **Conclusions**: The association between frequent physical activity and reduced mortality risk among Chinese older adults is profoundly mediated by baseline health status and functional capacity. These findings highlight the importance of integrated, multifactorial public health interventions that address chronic disease management and functional rehabilitation alongside physical activity promotion.

## 1. Introduction

China’s rapidly aging population has made the management of middle-aged and older adults’ health a pressing public health priority. According to the National Bureau of Statistics, by 2022, individuals aged 60 years or older accounted for 19.8% of China’s population, among whom approximately 30% experience chronic disease comorbidities [1]. Understanding the key determinants of mortality in this demographic is crucial for developing effective healthy aging strategies. While many factors contribute to longevity, modifiable health behaviors, sociodemographic characteristics, and baseline health status represent critical areas for investigation.

Among modifiable behaviors, physical activity has been extensively documented in international studies to have an inverse association with all-cause mortality [2,3]. For instance, a Korean cohort study demonstrated that participation in moderate-to-vigorous intensity aerobic exercise (≥50 MET-h per week) was linked to an 18% decrease in all-cause mortality and a 45% decline in cardiovascular mortality [4]. Often conceptualized as a form of “medicine,” physical activity has shown therapeutic efficacy for chronic disease management [5], including a notable protective effect against mortality in patients with chronic obstructive pulmonary disease (COPD) [6]. Emerging evidence also suggests that physical activity can promote broader health benefits by delaying physiological aging and enhancing quality of life [7]. However, these effects can be context-dependent and gender-specific. In one meta-analysis, high levels of recreational physical activity are significantly associated with reductions in all-cause and cardiovascular mortality among males with sedentary jobs [8], while among middle-aged and older women, regular walking and other recreational activities are strongly associated with reduced risks of all-cause mortality, cardiovascular disease, and stroke [9].

A substantial body of research confirms a dose–response relationship between physical activity and mortality, where benefits are observed across all intensity levels. For instance, studies show that achieving approximately 10,000 steps per day corresponds to a significant reduction in mortality risk [10]. Even minimal doses of moderate-to-vigorous physical activity are protective, with as little as 1–499 MET-min/week linked to a 22% lower risk [11]. Strikingly, even activity levels below recommended guidelines have yielded significant benefits; one study found that just 50 min of moderate activity per week reduced all-cause mortality by 31% in older adults [12]. Over the long term, sustained physical activity is associated with increased life expectancy, with estimates suggesting an extension of up to 4.5 years for moderate-intensity engagement [13]. Of particular clinical significance, physical activity also demonstrates protective associations against the incidence of at least 13 cancer types, most notably colorectal and breast cancers, through mechanisms hypothesized to involve the attenuation of systemic inflammation and enhancement of insulin sensitivity [14]. Conversely, insufficient physical activity remains a major contributor to the global burden of non-communicable diseases [15] and may indirectly increase mortality risk by promoting frailty and multimorbidity in older adults [16,17].

However, population-based cohort studies specifically targeting Chinese middle-aged and older adults remain scarce. Most existing research has examined only a single dimension of physical activity (e.g., frequency or intensity) [18], lacking a comprehensive analysis of its varied effects across complex health statuses and sociodemographic profiles.

Within the unique Chinese context, physical activity patterns differ significantly between urban and rural populations, with rural residents engaging more in farming labor while their urban counterparts rely on recreational activities like tai chi [19,20]. National data indicate that approximately 45% of this demographic do not meet WHO activity recommendations, often due to the burden of chronic diseases, functional decline, and inadequate social support [21,22]. Methodologically, many prior studies have used cross-sectional designs, which preclude a robust examination of the long-term, dynamic relationship between health behaviors and mortality. This approach often fails to elucidate the complex, reciprocal pathways between chronic disease, lifestyle factors, and the vicious cycle of “disability–reduced activity–deteriorating health” [23,24,25].

To address these limitations, this study leverages nationally representative longitudinal data to fill several critical gaps. First, it aims to provide the first investigation to examine the temporal association between physical activity frequency and mortality risk among Chinese middle-aged and older adults. Second, it will assess the complex interactions between health behaviors and socioeconomic determinants by incorporating them. Third, through stratified and sensitivity analyses, this study aims to pinpoint high-risk subgroups and provide more robust evidence for these populations.

Therefore, the aim of this study is to examine the association of a comprehensive set of factors—including physical activity frequency, sociodemographic characteristics, lifestyle behaviors, and baseline health status—with all-cause mortality in middle-aged and older Chinese adults. By sequentially adjusting for these variables, we seek to identify the most significant predictors of mortality and better understand the nuanced role that physical activity plays within the broader context of health and aging in China.

## 2. Data and Methods

### 2.1. Study Design

This study is a longitudinal cohort study based on the secondary analysis of data from the China Health and Retirement Longitudinal Study (CHARLS). The reporting of this study follows Strengthening the Reporting of Observational Studies in Epidemiology (STROBE) guidelines for cohort studies.

### 2.2. Data Source and Participants

This study analyzed longitudinal cohort data from the China Health and Retirement Longitudinal Study (CHARLS), a nationally representative survey tracking the health and socioeconomic status of middle-aged and older adults in China. Modeled after the Health and Retirement Study (HRS) in the United States, CHARLS aims to collect a comprehensive dataset from Chinese residents aged 45 and older. The baseline national wave was fielded in 2011, surveying approximately 17,500 individuals in 10,000 households across 150 counties/districts and 450 villages/resident committees, with follow-ups conducted every two years. The CHARLS survey employs a multistage stratified probability proportional-to-size (PPS) sampling design to ensure its representativeness across 28 provinces. The extensive questionnaire collects data on demographics, family structure, health status and functioning, biomarkers, health care, work and retirement, and income and assets.

### 2.3. Sample Size

We utilized multi-wave panel data spanning from 2011 baseline to 2020 follow-up, which included the Harmonized CHARLS Version D (Waves 1–4) and the 2020 follow-up (Wave 5). For this analysis, the inclusion criteria were (1) being a participant in the 2011 baseline wave of CHARLS, and (2) having complete data on baseline physical activity and follow-up mortality status. Participants with missing data on these key variables were excluded from the final analytic sample.

As this study involves the secondary analysis of a large-scale, pre-existing national dataset, a formal a priori sample size calculation was not performed. The sample size was determined by the number of eligible participants available within the CHARLS waves (2011–2020) who met the study’s inclusion criteria. From the initial CHARLS cohort, participants aged 45 years and older at the baseline wave were selected [26]. After excluding those with missing data on key variables, the final analytic sample was derived, with mortality status verified through official death registry records (*n* = 417) and household follow-up interviews (*n* = 1675). The resulting large sample provides sufficient statistical power for the multivariable Cox proportional hazards models conducted.

### 2.4. Outcome Variable

The primary outcome for this study was all-cause mortality. Mortality events were ascertained through official death registry records and verified during household follow-up interviews conducted through the 2020 wave.

### 2.5. Exposure Variable: Physical Activity

The primary exposure variable was physical activity frequency, quantified as the self-reported weekly count of days participating in moderate-to-vigorous intensity activities (e.g., walking, running, swimming, and bicycling). This variable was classified into three categories: (1) low frequency (≤1 day/week), (2) medium frequency (2–4 days/week), and (3) high frequency (≥5 days/week). It is important to note that the CHARLS questionnaire does not differentiate between leisure-time and occupational physical activity, which is a limitation of this study.

### 2.6. Covariates

A comprehensive set of covariates, selected based on the established literature, were included in the analysis. These encompassed three key domains: demographic characteristics (gender, age, marital status, education level, and household registration type [hukou]); lifestyle variables (smoking status, alcohol consumption, and body mass index [BMI] category); and health status indicators (limitations in activities of daily living [ADL], number of chronic conditions, and presence of serious illnesses at baseline).

### 2.7. Statistical Analysis

To prepare the data for survival analysis, the original wide-format dataset containing repeated measures was restructured into a long-format configuration. In this structure, each observation corresponded to an individual’s time-to-event record, where time zero (T0) was defined as the baseline interview date and follow-up continuing until either the date of death or the last known follow-up.

**Figure 1:**
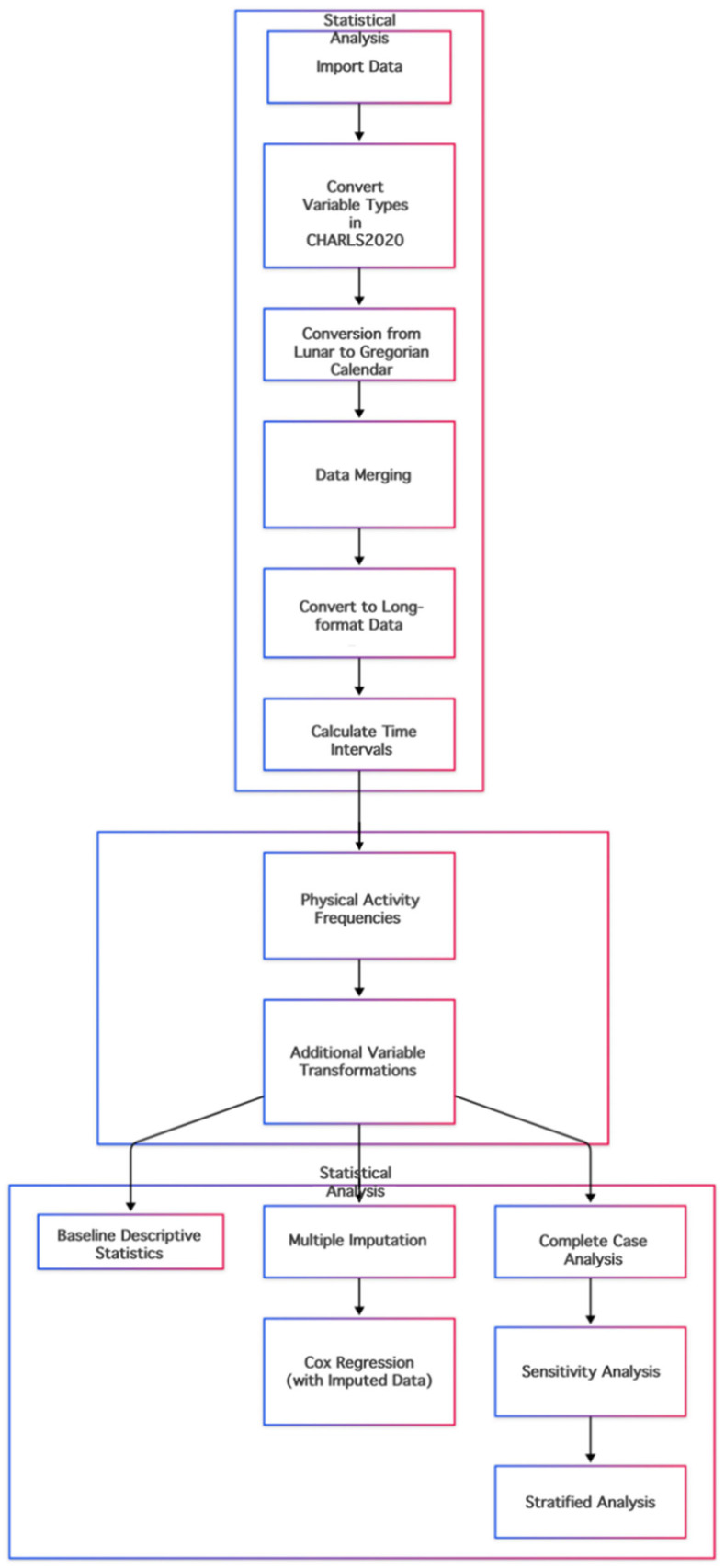
Variable cleanup flowchart.

Data preprocessing involved several key procedurals: (1) lunar calendar dates were standardized and converted to the Gregorian calendar, (2) survival time in months, (3) categorical variables were recoded into dummy variables, and (4) continuous, such as age, were categorized to address potential non-linearity. Finally, to handle missing data on covariates, we employed Multiple Imputation by Chained Equations (MICE), a robust approach for handling incomplete data in longitudinal studies. Figure 1 presents in detail the data processing procedure employed in this article.

All statistical analyses were implemented in R code (version 4.3.1) [27]. A *p*-value < 0.05 was considered statistically significant.

First, baseline characteristics of the study cohort were summarized using descriptive statistics. Group differences between the survival and mortality cohorts were evaluated using chi-square tests for categorical variables and independent t-tests or analysis of variance (ANOVA) for continuous variables. Effect sizes were calculated using Cramér’s V for categorical variables and Cohen’s d for continuous variables.

Second, survival probabilities were visualized using Kaplan–Meier curves, and cumulative mortality risk was visualized with Nelson–Aalen cumulative hazard curves. Log-rank tests were employed to assess for significant differences in survival distributions between the physical activity frequency groups.

Third, to examine the association between the exposure variables and all-cause mortality, we constructed a series of Cox proportional hazards regression models. This sequential approach allowed us to assess the influence of different domains of covariates. Model 1 was adjusted for demographic variables, including sex, age, marital status, education level, and household registration type. Model 2 additionally included lifestyle-related variables (e.g., smoking status, alcohol consumption, and body mass index [BMI]). Model 3, the fully adjusted model, further incorporated health status indicators, such as limitations in activities of daily living (ADL), number of chronic conditions, and presence of severe illness at baseline. Results from these models were reported as hazard ratios (HRs) with 95% confidence intervals (CIs), and model performance was assessed using likelihood ratio tests and the Akaike Information Criterion (AIC).

To test the robustness of our findings and explore potential effect modification, we conducted several secondary analyses. A sensitivity analysis was performed to minimize the risk of reverse causality by excluding participants who died within the first two years of follow-up (*n* = 124) and those with severe illnesses at baseline (*n* = 289). Furthermore, stratified analyses were conducted by age group (45–59, 60–74, and ≥75 years) and BMI category (underweight, normal weight, and overweight/obese). Interaction terms (e.g., physical activity × age group) were also included in the models to test for significant effect modification across these strata.

With R, multiple imputation for missing values was conducted using the MICE package [28]. Cox proportional hazards models were fitted using the coxph function from the survival package. Survival curves were generated and visualized using the ggsurvplot function from the survminer package [29].

## 3. Results

### 3.1. Baseline Characteristics of the Study Cohort

The baseline characteristics of the 2092 participants are presented in Table 1. Of these, 1675 were in the survival group and 417 were in the all-cause mortality group. The mean age of the total cohort was 63.7 (±10.4) years. The majority of participants were female (56.5%), married or cohabiting (90.7%), and resided in rural areas (81.8%). At baseline, 67.2% of the cohort reported having at least one chronic condition, and 19.4% reported limitations in activities of daily living (ADL).

Table 1 also provides a comparative assessment between the survival group (*n* = 1675) and the all-cause mortality group (*n* = 417). Significant group differences (*p* < 0.05) were observed for most baseline variables, including physical activity frequency, demographic characteristics, health status, and lifestyle factors.

Marked disparities in physical activity patterns were observed between the cohorts (*p* < 0.001). Specifically, 35.0% of individuals in the mortality group reported a low frequency of physical activity (≤1 day per week), a proportion substantially higher than the 8.1% observed in the survival group. Conversely, the proportion of individuals engaging in physical activity ≥5 days per week was significantly lower in the mortality group compared to the survival group.

Demographically, the mortality group was significantly older, with 41.7% aged ≥75 years compared to just 2.2% in the survival group (*p* < 0.001). A higher proportion of the deceased were also male (56.1% vs. 43.7%, *p* < 0.001) and unmarried (35.0% vs. 8.8%, *p* < 0.001).

The mortality group also carried a significantly higher burden of poor health. A larger proportion of the deceased had two or more chronic conditions (59.5% vs. 36.1%, *p* < 0.001) and reported ADL limitations (47.7% vs. 13.5%, *p* < 0.001). In terms of lifestyle and socioeconomic factors, the prevalence of current smoking and previous alcohol consumption was significantly higher in the mortality group (*p* < 0.001), and a larger proportion had a primary school education or less (94.0% vs. 89.2%, *p* = 0.005).

Finally, nutritional status, as measured by BMI, differed significantly between the groups (*p* < 0.001). The prevalence of underweight was substantially higher among the deceased, whereas the prevalence of overweight or obesity was greater in the survival group.

Significant disparities were also evident in lifestyle factors and socioeconomic characteristics between the two groups. The prevalence of current smoking and previous alcohol consumption was significantly higher among individuals in the mortality group (*p* < 0.001).

Regarding educational attainment, a significantly larger proportion of the mortality group had a primary school education or less (94%) compared to the survival group (89%, *p* = 0.005).

Regarding place of residence, 79% of the deceased resided in rural households, a proportion slightly lower than the 83% observed in the survival group. However, results from the multivariable analysis revealed that after adjusting for potential confounders, rural residency demonstrated a statistically significant protective association with mortality (HR = 0.54, 95%CI = [0.31, 0.94], *p* = 0.03).

### 3.2. Multivariable Cox Regression Analysis

The association between physical activity frequency and all-cause mortality among middle-aged and older adults was examined using a series of multivariable Cox proportional hazards models. The results revealed that the initially strong, protective association of physical activity was significantly attenuated and lost statistical significance after sequential adjustment for health status and lifestyle covariates. Table 2 displays the estimation results of the three regression models employed in this article.

In the minimally adjusted Model 1, which controlled only for demographic factors, physical activity frequency was significantly and inversely associated with mortality risk. Compared to the reference group engaging in physical activity ≤1 day per week, individuals who were active 2–4 days per week exhibited a 68% lower risk of death (HR = 0.32, 95% CI = [0.13, 0.81], *p* = 0.016), while those active ≥5 days per week showed a 67% reduction in mortality risk (HR = 0.33, 95% CI = [0.23, 0.49], *p* < 0.001).

However, after additional adjustment for baseline health status indicators in Model 2, this protective association was rendered non-significant. For individuals active 2–4 days per week, the hazard ratio increased to 0.64 (HR = 0.64, 95% CI = [0.18, 2.32], *p* = 0.5). For those active ≥5 days per week, statistical significance was no longer observed, and the hazard ratio increased to 0.73 (HR = 0.73, 95% CI = [0.38, 1.40], *p* = 0.30). In this second model, having limitations in activities of daily living (ADL) emerged as a strong and statistically significant predictor of mortality (HR = 2.20, 95% CI = [1.13, 3.15], *p* = 0.003).

In the full adjusted Model 3, which further controlled for lifestyle behaviors (e.g., smoking and alcohol consumption), the association between physical activity and mortality remained non-significant in the statistics. The hazard ratios for the medium- and high-frequency activity groups were 0.52 and 0.56, respectively (*p* > 0.05). In this final model, current smoking was identified as a strong, independent predictor of mortality, with a hazard ratio of 2.87 (HR = 2.87, CI = [1.44, 5.72], *p* = 0.003).

**Figure 2:**
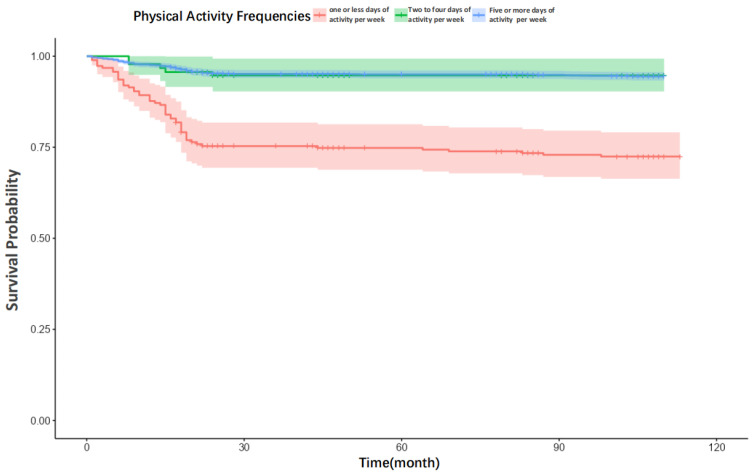
Survival curves for people with different physical activity frequencies.

With Kaplan–Meier survival curves (Figure 2), the group engaging in physical activity ≥5 days per week consistently exhibited the highest survival probability over the follow-up period, demonstrated by the most gradual rate of decline. In contrast, the group with ≤1 day of activity per week showed the steepest decline in survival. The survival curve for the 2–4 days per week activity group fell between these two extremes, trending more closely with the high-frequency group. During the initial follow-up, the survival probability for the most active group was approximately 15–20% higher than that of the least active group, an advantage that persisted through the middle of the follow-up period (5–10 years).

**Figure 3:**
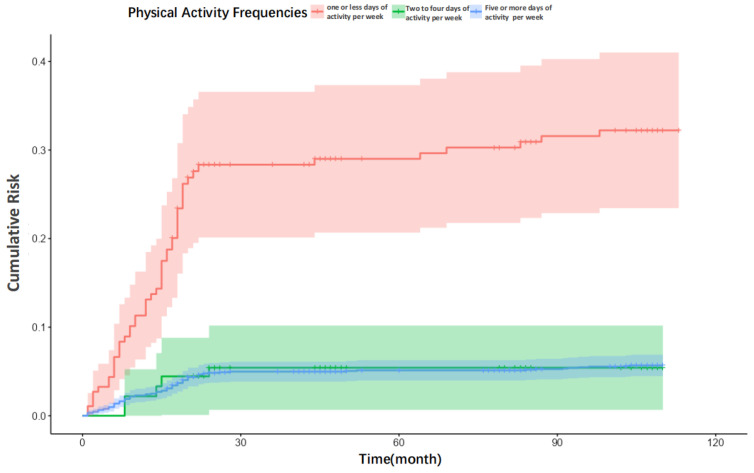
Cumulative risk curves for people with different physical activity frequencies.

The cumulative risk curves (Figure 3) further corroborate this pattern, illustrating the risk of all-cause mortality over time. The cumulative risk of death increased at the slowest rate in the group engaging in physical activity ≥5 days per week; the slope of the curve was significantly lower than that of the low-frequency activity group. For instance, by year five of follow-up, the cumulative risk for the most active group was approximately 40% lower than that for the group with ≤1 day of activity per week. By year ten, the difference expanded to approximately 60%. However, the difference in cumulative risk curves between the groups began to converge in the later stages of the follow-up period.

### 3.3. Sensitivity and Stratification Analysis

To assess the robustness of the findings, several sensitivity and stratified analyses were conducted. The results are presented in Table 3.

In a sensitivity analysis that excluded participants who died within the first two years of follow-up (*n* = 124), the hazard ratio (HR) estimates for physical activity frequency became unstable.

In a second sensitivity analysis that excluded participants with baseline cardiovascular or cerebrovascular diseases and cancer (*n* = 289), the association between high physical activity (≥5 days per week) and mortality risk was not statistically significant (HR = 0.68, 95% CI = [0.30, 1.54], *p* = 0.4).

Stratified analyses by BMI indicated that among individuals in the overweight/obese category, those who were active ≥5 days per week had a lower risk of mortality compared to the least active group, though this finding did not reach statistical significance (HR = 0.49, 95% CI = [0.16, 1.57], *p* = 0.2). In the normal-weight group, the hazard ratio for high physical activity was 0.83 (*p* = 0.8). When stratified by age, no significant association between physical activity frequency and mortality was found in any age group (*p* > 0.05 for all). The hazard ratio for high physical activity was 0.49 in the 60–74-year age group (*p* = 0.2) and was attenuated to 0.80 in the ≥75-year-old group (*p* = 0.7).

## 4. Discussion

In this large, nationally representative cohort study of middle-aged and older Chinese adults, we examined the complex interplay of physical activity, sociodemographic factors, lifestyle behaviors, and health status on all-cause mortality. Our principal finding is that while frequent physical activity shows a strong protective association with mortality in unadjusted models, this effect is significantly attenuated and loses statistical significance after accounting for baseline health status, particularly functional limitations. This suggests that the often-cited benefits of physical activity are not independent of an individual’s underlying health and functional capacity, a critical consideration given that our data, a noted limitation, could not distinguish between occupational and leisure-time activity. The analysis confirms that advanced age, current smoking, and limitations in activities of daily living (ADLs) are dominant, independent predictors of mortality. Conversely, being married and residing in a rural area were associated with a significant protective effect. These findings highlight the multifactorial nature of mortality risk in aging populations and underscore the critical role of health status as a mediator in the relationship between physical activity and longevity.

The attenuation of the physical activity effect after adjusting for health status is a key finding of this study. The initial strong protective effect (HR ≈ 0.33) aligns with a vast body of international literature demonstrating the benefits of exercise on metabolic regulation, immune competence, and cardiovascular health [2,3,5]. However, the loss of statistical significance in the fully adjusted model suggests that the benefits of physical activity are not independent of an individual’s underlying health. This supports the “disability–reduced activity–deteriorating health” cycle model [25], where poor health (e.g., chronic diseases, functional impairment) is a primary barrier to engaging in physical activity. In this vicious cycle, physical inactivity is both a consequence of poor health and a contributor to further decline. Therefore, while promoting physical activity is a crucial public health goal, our findings suggest that interventions may be less effective without concurrently managing the chronic conditions and functional limitations that prevent older adults from being active in the first place.

Consistent with the established literature, our analysis confirmed that advanced age and current smoking are among the most powerful predictors of mortality. The sharply increasing hazard ratio for older age groups reflects the cumulative burden of cellular senescence and age-related organ decline. The strong association between smoking and mortality underscores the well-documented pathophysiological damage caused by tobacco, including oxidative stress and vascular endothelial impairment [30]. The protective effect of being married also aligns with previous research, likely reflecting the multifaceted benefits of spousal support, which can include better health monitoring, economic stability, and reduced psychosocial stress.

Two findings from our study warrant a broader discussion. First, the observation that rural residence is protective against mortality (HR = 0.54 in the final model) is seemingly paradoxical, given the often-cited disparities in healthcare access between urban and rural China. While our initial hypothesis was that this might be due to higher levels of daily physical labor, this explanation is likely too simplistic. Other factors may be at play, including stronger social cohesion and informal support networks in rural communities, differences in diet, or lower levels of certain environmental pollutants and psychosocial stressors compared to urban centers [31,32,33]. Our study cannot disentangle these factors, highlighting the need for future research to explore the specific mechanisms behind the urban–rural mortality differential in China.

Second, our results contribute to the ongoing discussion of the “obesity paradox” in older adults. We found that both normal-weight and overweight/obese status were associated with a significantly lower mortality risk compared to being underweight. This finding does not necessarily contradict the known long-term risks of obesity. Instead, it may be explained by several factors prevalent in aging populations. One leading hypothesis is reverse causality, where chronic, undiagnosed illnesses cause unintentional weight loss, making a lower BMI a marker of underlying disease rather than a sign of health. Another explanation is the concept of metabolic reserve; in times of acute illness, having slightly higher adipose tissue reserves may provide a crucial energy buffer that aids survival. Finally, BMI is a crude measure that does not distinguish between fat mass and muscle mass. An older individual with a “normal” BMI may suffer from sarcopenia (low muscle mass), which is a strong predictor of frailty and mortality. This complex finding underscores the need to look beyond BMI alone and consider body composition and nutritional status when assessing mortality risk in older adults.

This study has several notable strengths. It utilizes a large, nationally representative longitudinal dataset (CHARLS) with a long follow-up period, allowing for a robust temporal analysis of mortality risk. The hierarchical modeling approach and the inclusion of a comprehensive set of multidimensional covariates allowed for a nuanced examination of the interactions between health behaviors and socioeconomic determinants. However, several limitations must be acknowledged. First, the physical activity data were self-reported and did not differentiate between occupational and leisure-time activity, which may have different health effects. This is a key limitation that prevents a deeper analysis of the “physical activity paradox.” Future studies should incorporate objective measures like accelerometry. Second, despite our comprehensive adjustments, the potential for residual confounding from unmeasured variables remains. Finally, the sample size in some subgroup analyses (e.g., the advanced age group) was limited, which may have affected statistical power and led to wide confidence intervals.

## 5. Conclusions

In conclusion, this study demonstrates that while frequent physical activity is associated with a lower risk of death among middle-aged and older Chinese adults, this relationship is profoundly mediated by baseline health status and functional capacity. The analysis of this large, nationally representative cohort—a key strength of the study—revealed that the most significant predictors of mortality are non-modifiable or deeply entrenched factors such as advanced age, chronic disease burden, and functional limitations. Our research offers empirical support for designing healthy aging strategies tailored to the Chinese population and contributes valuable evidence from a non-Western population to the broader field of global aging research.

However, a primary limitation of this study is its reliance on self-reported physical activity data, which did not distinguish between occupational and leisure-time activity. Future research using objective measures is needed to disentangle the health effects of different activity types.

Despite this limitation, our findings clearly underscore the need for integrated, multicomponent public health strategies. Promoting physical activity alone may be insufficient. To create an environment where healthy aging is possible, interventions must also focus on comprehensive chronic disease management and functional rehabilitation, particularly for the most vulnerable subpopulations.

## Figures and Tables

**Table 1:** Baseline descriptive statistics of the study cohort, stratified by mortality status.

Demographics Characteristic	Total Sample (*n* = 2092)	Ratio	Mortality Group (*n* = 417)	Ratio	Survival Group (*n* = 1675)	Ratio	*p*-Value
Age (mean SD)	63.7 (*SD* = 10.4)		70.9 (*SD* = 10.1)		61.9 (*SD* = 9.6)		<0.001
Gender							<0.001
Male	966	46.2%	234	56.1%	732	43.7%	
Female	1126	53.8%	183	43.9%	943	56.3%	
Marital Status							<0.001
Without Spouse	294	14.1%	147	35.3%	147	8.8%	
With Spouse	1798	85.9%	270	64.7%	1528	91.2%	
Education							0.005
Elementary or below	1886	90.2%	392	94.0%	1494	89.2%	
Secondary/High School	184	8.8%	21	5.0%	163	9.7%	
University or above	20	1.0%	2	0.5%	18	1.1%	
Residence							0.12
Urban (Non-Farm)	379	18.1%	87	20.9%	292	17.4%	
Rural (Countryside)	1712	81.9%	330	79.1%	1382	82.6%	
Health Status							<0.001
ADL Limitations	423	20.2%	196	47.0%	227	13.6%	
No. of Chronic Diseases							<0.001
0	615	29.4%	70	16.8%	545	32.5%	
1	624	29.8%	99	23.7%	525	31.3%	
≥2	853	40.8%	248	59.5%	605	36.1%	
Lifestyle Factors							
Physical Activity Freq. *n* (%)							<0.001
≤1 day/week	185 (8.8%)		50 (12.0%)		135 (8.1%)		
2–4 days/week	92 (4.4%)		6 (1.4%)		86 (5.1%)		
≥5 days/week	1815 (86.8%)		361 (86.6%)		1454 (86.8%)		
BMI Category							<0.001
Underweight (<18.5)	131	6.3%	54	12.9%	77	4.6%	
Normal (18.5–24.9)	747	35.7%	128	30.7%	619	37.0%	
Overweight/Obese (≥25)	889	42.5%	104	24.9%	785	46.9%	
Smoking Status *n* (%)							<0.001
Never	1292	61.8%	195	46.8%	1097	65.5%	
Former	173	8.3%	61	14.6%	112	6.7%	
Current	466	22.3%	120	28.8%	346	20.7%	
Alcohol Consumption							<0.001
Never	1236	59.1%	230	55.2%	1006	60.1%	
Former	194	9.3%	78	18.7%	116	6.9%	
Current	553	26.4%	105	25.2%	448	26.7%	

Data are presented as *n* (%) for categorical variables and mean (*SD*) for continuous variables. *p*-values were derived from Pearson’s Chi-squared test for categorical variables and an independent t-test for continuous variables.

**Table 2:** Multivariable Cox regression analysis for all-cause mortality.

Variable	Model 1 ^1^		Model 2 ^2^		Model 3 ^3^	
	HR (95% CI)	*p*-Value	HR (95% CI)	*p*-Value	HR (95% CI)	*p*-Value
Physical Activity Freq.						
≤1 day/week (Ref)	—	—	—	—	—	—
2–4 days/week	0.32 (0.13, 0.81)	0.016	0.64 (0.18, 2.32)	0.5	0.52 (0.14, 1.91)	0.3
≥5 days/week	0.33 (0.23, 0.49)	<0.001	0.73 (0.38, 1.40)	0.3	0.56 (0.29, 1.09)	0.089
Gender						
Male (Ref)	—	—	—	—	—	—
Female	0.69 (0.48, 0.98)	0.038	1.78 (0.88, 3.59)	0.11	1.53 (0.76, 3.07)	0.2
Age Group (years)						
45–59 (Ref)	—	—	—	—	—	—
60–74	2.85 (1.75, 4.61)	<0.001	2.32 (1.24, 4.33)	0.008	2.20 (1.17, 4.13)	0.014
≥75	15.2 (8.96, 25.9)	<0.001	15.1 (7.27, 31.6)	<0.001	15.1 (7.25, 31.3)	<0.001
Marital Status						
Without Spouse (Ref)	—	—	—	—	—	—
With Spouse	0.56 (0.37, 0.83)	0.004	0.53 (0.30, 0.94)	0.030	0.56 (0.32, 1.00)	0.050
Education						
Elementary school and below	—	—	—	—	—	—
Junior high school/high school/technical secondary school	0.93(0.44, 1.99)	0.9	1.34 (0.54, 3.31)	0.5	1.41 (0.57, 3.5)	0.5
University and above	0.00 (0, Inf)	>0.9	0.00 (0, Inf)	>0.9	0.00 (0, Inf)	>0.9
Residence						
Urban (Ref)	—	—	—	—	—	—
Rural	0.73 (0.48, 1.09)	0.12	0.48 (0.28, 0.85)	0.012	0.54 (0.31, 0.94)	0.030
ADL Limitations						
None (Ref)	—	—	—	—	—	—
Present	—	—	2.20 (1.31, 3.69)	0.003	1.89 (1.13, 3.15)	0.015

HR = Hazard Ratio; CI = Confidence Interval; Ref = Reference Group. ^1^ Model 1: Adjusted for demographic variables (gender, age, marital status, education, and residence). ^2^ Model 2: Model 1 + health status (ADL limitations). ^3^ Model 3: Model 2 + lifestyle factors (smoking, alcohol consumption—not shown in table for brevity).

**Table 3:** Sensitivity and stratified analyses of physical activity on all-cause mortality.

Analysis Group and PA Frequency	HR (95% CI)	*p*-Value
Sensitivity Analysis		
Exclude deaths < 2 years		
≤1 day/week (Ref)	—	—
2–4 days/week	Unstable ^1^	>0.9
≥5 days/week	Unstable ^1^	>0.9
Exclude baseline CVD/cancer		
≤1 day/week (Ref)	—	—
2–4 days/week	0.70 (0.18, 2.78)	0.6
≥5 days/week	0.68 (0.30, 1.54)	0.4
Stratified by BMI		
Underweight		
≥5 vs. ≤1 day/week	0.40 (0.09, 1.75)	0.2
Normal weight		
≥5 vs. ≤1 day/week	0.83 (0.25, 2.72)	0.8
Overweight/Obese		
≥5 vs ≤1 day/week	0.49 (0.16, 1.57)	0.2
Stratified by Age (years)		
45–59		
≥5 vs. ≤1 day/week	0.58 (0.11, 2.99)	0.5
60–74		
≥5 vs. ≤1 day/week	0.49 (0.18, 1.35)	0.2
≥75		
≥5 vs. ≤1 day/week	0.80 (0.22, 2.90)	0.7

All models are fully adjusted. HR = Hazard Ratio; CI = Confidence Interval; Ref = Reference Group; PA = Physical Activity; CVD = Cardiovascular Disease. ^1^ Unstable estimate due to small sample size in the subgroup. For brevity, only the comparison between the highest (≥5 days/week) and lowest (≤1 day/week) PA frequency is shown for stratified analyses.

## Data Availability

The data used in this study primarily come from the following databases: Green patent data are sourced from the China Health and Retirement Longitudinal Study (CHARLS).
